# Design and Development of a Person-Centered Patient Portal Using Participatory Stakeholder Co-Design

**DOI:** 10.2196/11371

**Published:** 2019-02-11

**Authors:** John Kildea, John Battista, Briana Cabral, Laurie Hendren, David Herrera, Tarek Hijal, Ackeem Joseph

**Affiliations:** 1 Gerald Bronfman Department of Oncology McGill University Montreal, QC Canada; 2 Medical Physics Unit McGill University Montreal, QC Canada; 3 Cancer Research Program Research Institute of the McGill University Health Centre Montreal, QC Canada; 4 Department of Biomedical Engineering McGill University Montreal, QC Canada; 5 Division of Radiation Oncology McGill University Health Centre Montreal, QC Canada; 6 School of Computer Science McGill University Montreal, QC Canada; 7 Department of Medical Physics McGill University Health Centre Montreal, QC Canada

**Keywords:** patient portals, patient participation, telemedicine, software design

## Abstract

**Background:**

Patient portals are increasingly accepted as part of standard medical care. However, to date, most patient portals provide just passive access to medical data. The use of modern technology such as smartphones and data personalization algorithms offers the potential to make patient portals more person-centered and enabling.

**Objective:**

The aim of this study is to share our experience in designing and developing a person-centered patient portal following a participatory stakeholder co-design approach.

**Methods:**

Our stakeholder co-design approach comprised 6 core elements: (1) equal coleadership, including a cancer patient on treatment; (2) patient preference determination; (3) security, governance, and legal input; (4) continuous user evaluation and feedback; (5) continuous staff input; and (6) end-user testing. We incorporated person-centeredness by recognizing that patients should decide for themselves their level of medical data access, all medical data should be contextualized with explanatory content, and patient educational material should be personalized and timely.

**Results:**

Using stakeholder co-design, we built, and are currently pilot-testing, a person-centered patient portal smartphone app called Opal.

**Conclusions:**

Inclusion of all stakeholders in the design and development of patient-facing software can help ensure that the necessary elements of person-centeredness, clinician acceptability, and informatics feasibility are achieved.

## Introduction

### Context

A patient portal, in its most basic form, is a secure extension of a health care institution’s electronic medical record (EMR) that is accessible to patients [1,2]. It provides patients with access to some or all of their personal health information (PHI) within the EMR. Patient portals are generally positively reviewed by patients and clinicians and have been associated with improved patient engagement, patient empowerment, and patient satisfaction [3-11].

The argument for sharing PHI with patients via a patient portal is clear. However, the best way to do so is not obvious [12,13]. According to Prey et al [14], similar barriers to enabling patient access to clinical data are encountered throughout the world, highlighting the importance of reporting strategies used for patient portal design, development, and adoption. Despite this, few reports describing how patient portals were designed and developed and few detailed evaluations of their usability are available in the literature to provide guidance for creating new software.

Furthermore, in the present era of mobile devices and using more advanced computing technologies, patient portals can offer much more than just passive sharing of PHI with patients via the Web [15]. They can provide personalized educational material that explains the PHI, the patient’s condition, and treatment options; waiting room tools such as mobile check-in with call-in for appointments and waiting time estimates; communication tools such as patient-reported outcome (PRO) questionnaires and secure patient-clinician messaging; and other assisting functionalities on a variety of devices, including smartphones. As such advanced patient portals account for the fact that patients (care recipients) are people with complex needs that extend beyond just the immediate delivery of care, and as described in Rigby et al [15], they help equalize the patient-clinician knowledge balance by incorporating the patient as an equal member of their own care team, we will refer to them as *person-centered patient portals*. Person-centered needs include the ability to plan ahead and know one’s position in a waiting list, to feel in control of one’s own care, to understand one’s treatment options, and to share in all decision making about one’s care.

With all of the above in mind, this paper describes the approach we used to design and develop a person-centered patient portal, which we call *participatory stakeholder co-design*, involving patients and health care providers. The resulting patient portal, called Opal [16] represents a real-world example of a patient-facing electronic health (eHealth) project that was designed and built from scratch within the health care system. Opal supports personalized information, including PHI with appropriate explanations, appointment schedules with appointment-specific advice, mobile appointment check-in and call-in functionality, questionnaires, educational material automatically tailored to health condition and treatment plan, and an autonomous module specific to an institution’s patients’ committee. In our estimation, the success of the Opal project to date is due in large part to our participatory stakeholder co-design approach. Success in this context is measured in terms of the project’s ability to go from conception through design and development to use by patients in a pilot release, having navigated and survived the various legal, logistical, and cultural hurdles thrown up by the health care system.

### Background

As is the case for most health care technology, the development of a person-centered patient portal is complex, involving many stakeholders and numerous organizational layers that each may determine the success or failure of the initiative [15,17-19]. Numerous authors have pointed out the importance of employing user participatory design in software development [20-22], including eHealth projects [23-28] such as patient portals [29-32]. Indeed, according to a frequently cited report by the Standish Group International [33], user involvement in the design process is the number one reason why software projects succeed or, conversely, the lack thereof is the main reason why projects fail.

In the case of design of a person-centered patient portal, as was the aim of this study, the patient is the user, and patient involvement brings the design process into the realm of *patient co-design*, which itself is increasingly recognized as an integral component of sustainable quality improvement in health care [34-36].

Patient *co-design* is often confused and conflated with *patient-centered design* and *person-centered design*. However, these 3 concepts have distinctly different meanings [34,37,38], and it is worth stressing the difference between them. In *co-design*, patients help identify the process or project that needs to be designed (or redesigned) based on their personal experience, and they codrive the effort in partnership with the clinical team. In effect, as we learned from the presently described project, the effort is really *participatory stakeholder co-design*, our preferred term, since both patients and clinicians are equal stakeholders in the final result and they actively participate in all aspects of the design process. In *patient-centered* design, the design team strives to ensure that the needs of the patient are centermost. However, the project being designed may or may not have been identified by patients, and the design process may or may not involve actual patients. Similarly, in *person-centered design*, the design team works to ensure that the needs of the patient, as a whole person and an equal partner in their care [37], rather than simply a passive recipient, are foremost. Again, however, the design process may be person-centered without having the patient in the room and fully involved in the project. The design process is only *participatory stakeholder co-design* if all stakeholders are fully and equally involved.

In the context of a person-centered patient portal, stakeholder co-design ensures that the necessary elements of person-centeredness, clinician acceptability, and informatics feasibility are accounted for, with each element being necessary for the ultimate success of the portal.

As described in this paper, we believe that the uniqueness of the Opal patient portal is that it was designed from the ground up, in a noncommercial environment, to be person-centered using a participatory stakeholder co-design approach.

### Setting

Our center, the Cedars Cancer Centre, is a new comprehensive cancer center within the McGill University Health Centre (MUHC), a large academic teaching hospital with an affiliated Research Institute (RI-MUHC) in Montreal, Quebec, Canada. The Cedars Cancer Centre was formed in 2015 when the previously disparate cancer services (radiotherapy, medical oncology, surgical oncology, and supportive care) of the MUHC were brought together under one roof.

Our patient portal development team followed our participatory stakeholder co-design approach from the very beginning. The team was formed in 2014 when LH (who is also a professor of computer science) was receiving radiotherapy for breast cancer under the direction of radiation oncologist TH. Following a conversation regarding the computational needs of radiation oncology, TH introduced LH to JK, a medical physicist with whom he was already collaborating on a number of custom eHealth projects. The 3 decided to colead a health informatics research collaboration, with a vision to use participatory stakeholder co-design to improve the experience and outcomes of people receiving care at our cancer center. The 3 coleads (patient who happens to be a computer scientist, radiation oncologist, and medical physicist) have led the research effort as equals since the beginning, each bringing their own unique but complementary expertise to the leadership table. LH, as a patient, was able to provide the patient perspective and access to other patients through the patients’ committee of the cancer center. LH, as a computer scientist, was able to contribute computational expertise and access to computer science students. JK, as an academic medical physicist, was able to provide health informatics know-how, facilitate access to the EMR, and access to medical physics students. TH, as a radiation oncologist and acting chief of the Division of Radiation Oncology, was able to provide his clinical expertise, help identify the needs and concerns of staff, manage necessary change within radiation oncology, and provide access to senior hospital management.

Overall, 2 areas for improvement were quickly identified by LH from her perspective as a person who was experiencing cancer treatment: (1) the *pain* of waiting for health care services and (2) the lack of PHI and relevant educational material provided to patients. A potential solution to first area of improvement was proposed in the form of a *patient app* to provide patients with personalized waiting time predictions determined using timestamp data and machine learning algorithms. Institutional funding was secured and the project began in the summer of 2015. As discussions around the functionality of the patient app evolved, it became clear that it could also serve as a patient portal and, as such, it could address second area of improvement, the lack of PHI and relevant educational material provided to people receiving care at the institution.

### Objectives

Opal represents a real-world example of a patient-facing eHealth initiative that was designed and developed from within the health care system using an approach that we have called *participatory stakeholder co-design*, with a cancer patient who is also a computer scientist, clinician, and medical physicist equally coleading the effort. Our participatory stakeholder co-design approach was fundamental to the success of the software from all perspectives—patient, clinician, and informatics. The purpose of this paper is to share how we used this participatory stakeholder co-design approach, and the key elements that we identified within our approach, to create a patient portal that we believe is both person-centered and useful.

## Methods

### Context

At the outset of this project, a patient portal was not available either within our institution or within Quebec’s public health care system. We scanned the commercial marketplace but were not able to identify an existing solution that provided our most basic need—multilingual support (French and English)—nor our person-centeredness requirements such as waiting room management tools (needed to reduce the pain of waiting), automated access to medical notes within any EMR, and automated personalization of information delivery. Accordingly, a custom-developed solution was pursued.

### Insights From the Literature

We studied the literature pertaining to the design of a patient portal, including the typical features that portals contain, the PHI elements that they offer, and the layouts that are used. We were particularly interested in publications detailing patient portals in oncology and for smartphones (our initial focus), but our search was not limited to these areas. Our intention was to inform ourselves regarding previous work, recommended best practices, areas where we could improve upon using our participatory stakeholder co-design approach, and challenges that our approach might address. To remain updated, our literature search was repeated many times over the course of the project.

We found 3 reports in the literature that provided particularly useful guidance on the presentation of patient data:

- Ahern et al [39] split patient portal services into 3 main categories: (1) information and transactions, (2) expert care, and (3) self-care and community. Transactions are bureaucratic in nature, such as viewing or creating or changing appointments, filling out forms, and requesting information. Expert care includes access to clinical services such as secure messaging, remote monitoring, and PRO questionnaires with clinician feedback. Self-care and community relates to relevant educational material and access or referral to services and social networks that provide support.
- A study by Tang et al [40] contains definitions and recommendations regarding data that an Electronic Health Record-tethered Personal Health Record (essentially a patient portal) should contain. Of note, it points out that to be useful to patients, health data should be accompanied by tools that help the patient understand and act on them, and data presentation should be adapted to the individual to optimize potential benefits.
- Baudendistel et al [12] point out how patients want to be able to track their long-term medical history and that the information provided must be accessible and filtered to the patient’s specific situation. Furthermore, they found that both patients and providers found value in PROs.

We were unable to find any detailed literature on the design of mobile patient portal apps.

### Participatory Stakeholder Co-Design

**Figure 1:**
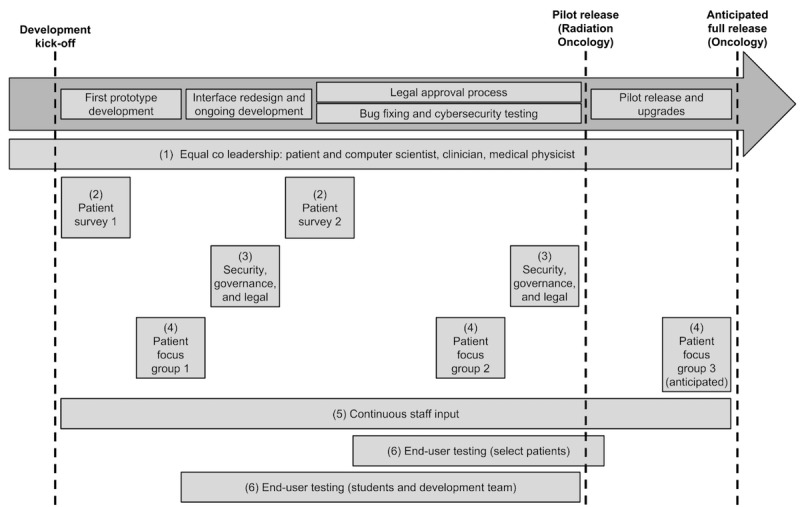
Approximate timeline for the Opal project showing how the 6 elements of our participatory stakeholder co-design methodology came together, culminating in the pilot release of Opal in Radiation Oncology. Each element is numbered in the figure and explained in the text. Just over 3 years of co-design and development were undertaken between the development kick off (May 2015) and the pilot release (June 2018). Figure not to scale.

Our methodology for participatory stakeholder co-design comprised 6 key elements. These included (1) equal coleadership (patient who is also a computer scientist, clinician, and medical physicist); (2) patient preference determination; (3) security, governance, and legal input; (4) user evaluation and feedback; (5) continuous staff input; and (6) end-user testing. Figure 1 presents an approximate timeline for the Opal project showing how the various elements of our participatory stakeholder co-design methodology came together, culminating in the pilot release of the Opal smartphone app in Radiation Oncology at the Cedars Cancer Centre. We elaborate on each of the 6 elements below.

#### Element 1: Equal Coleadership

Our 3 coleads partnered in leading all aspects of the design and development process. At a practical level, this meant that all 3 were equally involved in all important decisions and in constant communication, usually by email, videoconference, or in person. Patient participation was facilitated by a parking pass (provided by virtue of our patient colead being part of the cancer center’s patients’ committee) and by associate membership of the institution’s research institute (which also allowed LH to officially work on the project during her sabbatical year) but was complicated by disease progression and treatment. Videoconferencing was vital to ensuring full patient participation at all meetings.

#### Element 2: Patient Preference Determination

To obtain input from the wider population of people receiving cancer treatment (our initial focus), we conducted a voluntary convenience sampling survey within the waiting rooms of our cancer center. Participants were asked to provide basic demographic information (age and gender), state whether or not they use a smartphone, and if they would avail of a patient portal to access their PHI. A number of possible patient portal features were presented, and respondents were asked to rate their interest in having each using a 5-point Likert scale, ranging from “not at all interested” to “very interested.” Patients were invited to complete the main part of the survey only if they had reported that they use a smartphone.

To determine participants’ level of comfort with accessing their PHI, we listed 3 possible levels of PHI and asked them to select which one they would choose if given the choice. The 3 levels included:

1. I would like access to all of my medical record, including lab results, as soon as the information is available.
2. I would like access to all of my medical record, including lab results, after I have reviewed them with my doctor.
3. I would like access to just my appointment schedule and other need-to-know information.

We note that our survey was conducted after the development of our portal had begun and that it was conducted during 2 summer periods (with the help of summer students). The goal of the survey was to affirm patient preferences and verify that our software design was on the right track by sampling the wider patient population.

#### Element 3: Security, Governance, and Legal Input

Early into the development process (about 6 months), when a sufficiently developed prototype was available for demonstration, we engaged our institution’s Security and Governance team to provide guidance regarding the security and confidentiality of patient data and compliance with applicable regulations. Guidance was provided by means of a number of in-person meetings, email correspondence, and ultimately, a detailed risk assessment report.

The technical cybersecurity aspects of the software were validated by an internal vulnerability assessment by the institution’s Information Services security team and gray box penetration testing by an independent external consulting firm. Legal support (drawing up agreements and disclaimer forms) was provided by the institution’s Legal team and the Business Development office of our affiliated research institute.

#### Element 4: User Evaluation and Feedback

Once a prototype version of our patient portal smartphone app was ready, we invited a number of patients to participate in a purposeful sample focus group to provide feedback regarding its features and usability. Our radiation therapy team helped us to identify a number of engaged patients who had finished their treatments. A total of 10 patients were identified and contacted and 3 ultimately participated. Those who declined to participate indicated that they were unavailable at the time and date chosen.

Without describing the smartphone app or divulging its features, we started the focus group by asking participants for their thoughts on what information they would like to see in a smartphone portal without regard to the technical, legal, or logistical challenges on providing such information (ie, in an ideal world scenario). We then demonstrated the prototype app and observed the participants using it. Finally, we went through each of the features of the prototype and sought feedback. Our focus group guide was developed by the project leadership and was designed to solicit maximum feedback about the prototype app and the ease with which patients were able to navigate its features. A redacted version of our guide is provided in Multimedia Appendix 1.

Shortly before the pilot release, we conducted a second focus group comprising 5 members of the cancer center’s patients’ committee. The goal of this focus group was to rehearse the registration process and anticipate initial real-world problems and questions.

#### Element 5: Continuous Staff Input

Continuous staff input was ensured by our clinician colead. In addition, a radiation therapist (JB) participated in the design team during the first year of development. Over the course of the project, we also presented the planned features and functionality of the smartphone portal to various staff groups, ranging from on-the-floor care providers to senior management and the board of directors of the institution. The purpose of these presentations was threefold: (1) to ensure awareness and build buy-in at all levels, (2) to obtain staff feedback and address staff concerns, and (3) to seek support to continue development beyond the initial pilot release. During the final 2 months before the pilot release, a content development team comprising 2 radiation therapists, 2 medical physicists, an oncology nurse, a radiation oncologist, and an administrative assistant met weekly to ensure that the content of the app and the education material were *production ready*.

#### Element 6: End-User Testing

To help ensure usability and to identify bugs, we engaged volunteer testers as soon as the first prototype was ready. Overall, 2 types of volunteers were recruited: student testers (approximately 50 students in total) who used mock patient accounts, and real patients (including our colead) who had limited access to their PHI but full access to appointment schedules, waiting room management functionality, and available educational material. Our volunteer end-user testers provided invaluable feedback.

### Development Approach

We employed an *Agile* development approach [41] in which we rapidly prototyped various features and iteratively integrated and tested them in the main product. Our approach was facilitated by the research environment of our academic hospital and our strong links with McGill University. Over 3 summers, we employed summer students to develop prototypes of various features and we engaged undergraduate and graduate students (typically 7-10 computer science and medical physics students) in term research projects to explore possible new features. A core team of developers worked on the official version of the smartphone app for pilot release. The core team met at least weekly (but generally more frequently) with the coleads to discuss user feedback and bug fixes and communicated daily in person and via email. During term, student meetings were held roughly once every 2 weeks. The Github Issues tool [42] was used to submit bugs, the Zenhub board [43] was used to track them, and the Crashlytics platform [44] was used to release frequent builds to volunteer testers. At the time of pilot release, the core team comprised 3 full-time developers (1 front end, 1 back end, and 1 full stack) and 2 part-time full-stack developers (2 days per week each, typically).

### Pilot Release

A pilot release of our smartphone patient portal app, involving patients receiving radiotherapy at our center, is currently underway. We are taking a phased approach to the pilot, starting slowly with a small number of invited patients under the care of specific physicians. Our goal is to expand gradually to include all radiation oncology patients within 3 months, followed by a full release to all patients at our cancer center. Results of the pilot study will be submitted for publication once complete.

## Results

In this section, we present the results of our design and development research project that gave rise to Opal as a person-centered patient portal, and we detail the contributions provided by each element of our participatory stakeholder co-design methodology.

### Participatory Stakeholder Co-Design

#### Element 1: Equal Coleadership

Equal coleadership ensured that the main stakeholder perspectives of the project were always represented at the leadership table. In turn, these perspectives ensured that the design and development of Opal incorporated the necessary elements of person-centeredness, clinician acceptability, and informatics feasibility. A nonexhaustive list of these elements, as identified over the course of the project, is provided in Multimedia Appendix 2.

Although full and equal coleadership was a fundamental element of our participatory stakeholder co-design approach, we found that this was often not clear to people outside of the immediate design and development team. External to the team, it was necessary to continuously insist that all 3 coleads were equal. The tendency in the hierarchical health care system is to assume that a project must be led by a clinician, and it is commonly assumed that the patient participant is a token member of the team. Overcoming this required persistence, it ensured that the team was truly coled and it broke new ground within the hospital by demonstrating the benefits of full and equal patient involvement. Equal coleadership was vital to the success of the project.

#### Element 2: Patient Preference Determination

**Figure 2:**
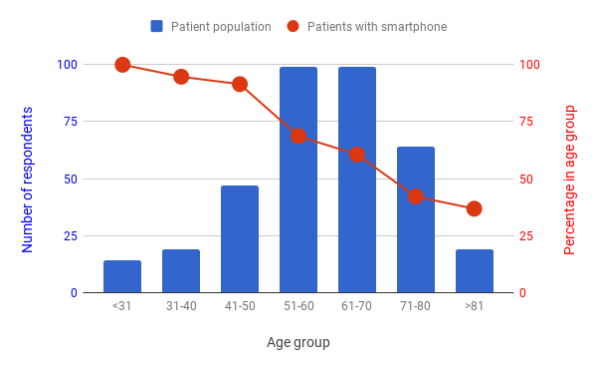
Distribution of the ages and smartphone usage of the 361 cancer patients who participated in our waiting room survey. Overall, 66% (237) of the respondents reported that they use a smartphone. These data demonstrate that a smartphone app would reach a broad patient population. The survey was conducted during the summer of 2016 and repeated during the summer of 2017 in the waiting rooms of our cancer center.

A total of 361 patients participated in our voluntary convenience-sampled survey. Of these, 65.7% (237/361) said they did have a smartphone. Figure 2 presents the distribution of patients by age group and the percentage of patients in each age group who reported having a smartphone. It addresses a concern we heard often that many older cancer patients may not have smartphones. As expected, smartphone usage is lower for the older age groups. However, it is clear that a smartphone app would nevertheless have uptake across the age spectrum and so can be considered a potentially useful tool for all patients. We also note that many older patients come to appointments with younger caregivers.

**Figure 3:**
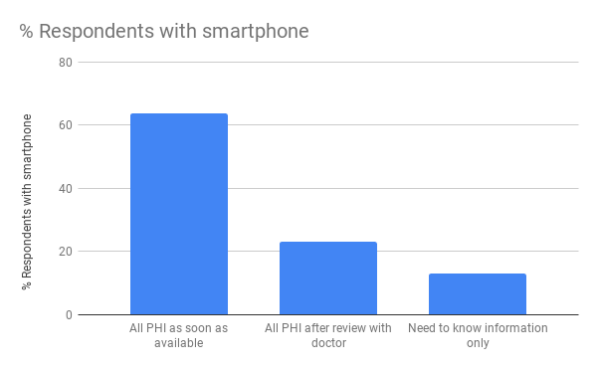
Patient preferences with regard to the personal health information (PHI) that they would like to access via an app or portal. We only included responses from patients who reported that they had a smartphone and who selected just 1 of the 3 options (n=232).

With regard to patient preferences for access to their PHI using an app or portal, we found that the majority of respondents with a smartphone (63.8%, 148/232) would prefer to have access to all of their data immediately once they are available. The remainder of respondents were roughly split two-to-one in favor of accessing all their data after review with their physician or accessing just need-to-know information such as appointments and educational material. Figure 3 shows the breakdown of patient’s PHI access preferences.

Table 1 presents the results of the main part of the survey and shows that the vast majority of patient respondents indicated that they were very interested in all of the possible features of an app or portal that we presented.

#### Element 3: Security, Governance, and Legal Input

The risk assessment report provided by our institution’s Security and Governance team comprised 16 specific recommendations with regard to data security and confidentiality. As they may be of use to other teams developing patient-facing software, we have summarized these recommendations in Multimedia Appendix 3.

In our experience, the legal aspect of the project, which was mainly beyond our control, was the slowest and most frustrating component. The main time-consuming legal issues encountered pertained to ownership of intellectual property, liability, and the contents of the patient disclaimer form. As the health care system is a busy legal environment with many competing priorities, we found that our nonurgent legal needs took time to be completed and required constant follow-up. Access to a separate legal team for business development tasks was not possible but would have helped.

#### Element 4: User Evaluation and Feedback

**Figure 4:**
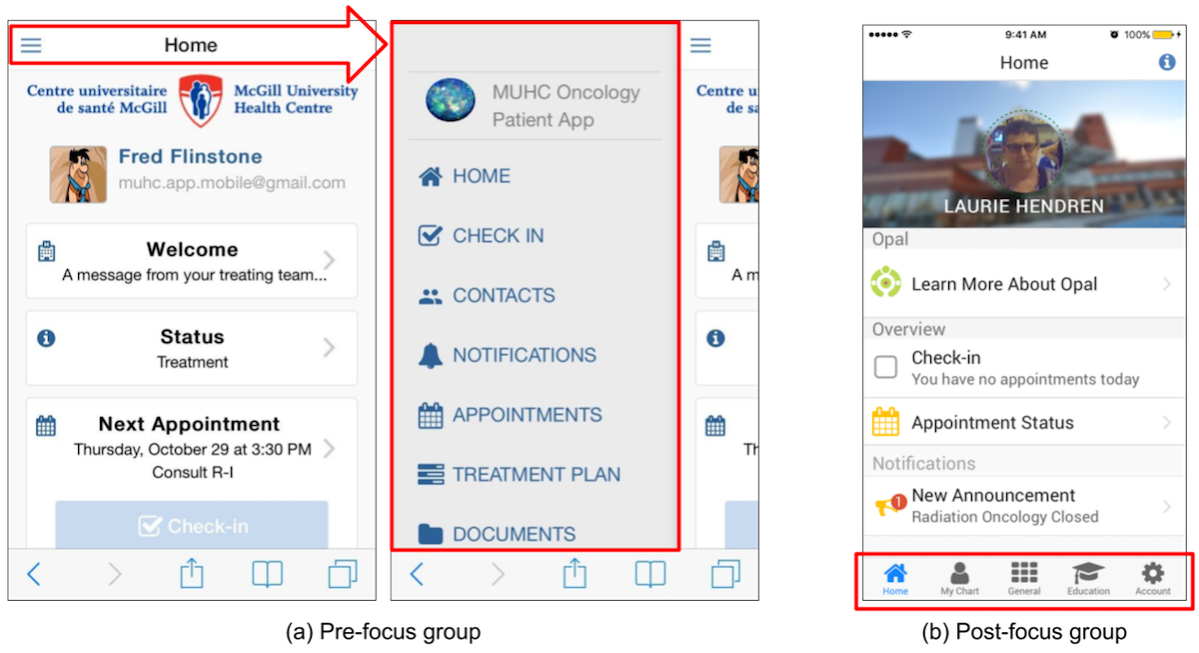
Screenshots of the Opal app demonstrating how the user interface changed based on feedback received during the first focus group. (a) The pre-focus group hamburger menu (illustrated by the red arrow and outlined section) was replaced with (b) a simpler and more intuitive bottom-of-the-screen tab-based menu.

Most of the features suggested by the participants at the start of the first focus group were already in our early prototype. These included hospital maps, radiotherapy treatment plan views with beam entry points, appointment schedules, waiting time estimates, and notifications. Some suggestions, such as an in-built feature to pay for hospital parking or a system to leave questions for call back (rather than navigating the hospital’s phone system), were not possible to include in the first release but will be included in future versions. Participants found the initial user interface difficult to understand. In particular, the “hamburger” side menu that was used to provide access to the options available within the app was nonintuitive and needed to be explained. This finding provoked a complete redesign of the user interface. Figure 4 illustrates the evolution of the user interface based on focus group feedback.

Interestingly, we found that our focus group participants prefaced many of their responses with disclaimers such as “I doubt it is possible, but it would be nice if...” or “I wouldn’t want to disturb my treating team by asking for this information, but it would be nice if I could see it myself in my own time.”

Our second focus group was quite different to the first as the product was mature (pilot-release ready) and participants accessed their actual data. The focus group’s main purpose was to evaluate the registration workflow, but it also provided important feedback regarding data and content. As the pilot release was centered in radiation oncology and only partially integrated with the medical oncology databases, patients receiving both radiotherapy and chemotherapy were confused that not all of their data were accessible. This motivated the development team to expand the pilot beyond radiation oncology as soon as possible.

**Table 1:** Results from the main part of the patient survey regarding possible features of a patient app or portal. Participants were presented with possible features of an app or portal and asked to rate their interest in having them using a 5-point Likert scale, ranging from “1=not at all interested” to “5=very interested”.

Possible feature	1, n (%)	2, n (%)	3, n (%)	4, n (%)	5, n (%)	*Pos^a^*, n (%)
Your personal appointment schedule (N=267)^b^	11 (4.1)	9 (3.4)	13 (4.9)	21 (7.9)	213 (79.8)	234 (87.6)
Secure access to doctor’s notes in your medical record (N=269)	20 (7.4)	11 (4.1)	9 (3.3)	33 (12.3)	196 (72.9)	229 (85.1)
Secure access to your personal laboratory results (N=267)	21 (7.9)	8 (3.0)	12 (4.5)	26 (9.7)	200 (74.9)	226 (84.6)
Educational material specific to your diagnosis (N=259)	16 (6.2)	7 (2.7)	17 (6.6)	54 (20.8)	165 (63.7)	219 (84.6)
Educational material specific to your phase of treatment (N=259)	19 (7.3)	9 (3.5)	15 (5.8)	54 (20.8)	162 (62.5)	216 (83.4)
Notifications sent to your phone to advise you that you are next in line to see your doctor or for treatment (N=267)	25 (9.4)	13 (4.9)	8 (3.0)	26 (9.7)	195 (73.0)	221 (82.8)
Personalized check-in and call-in for your appointments via your phone (N=269)	26 (9.7)	7 (2.6)	18 (6.7)	34 (12.6)	184 (68.4)	218 (81.0)
Contact information for your treating team (N=260)	21 (8.1)	10 (3.8)	21 (8.1)	37 (14.2)	171 (65.8)	208 (80.0)
A secure messaging system with your treatment team (N=262)	21 (8.0)	11 (4.2)	20 (7.6)	34 (13.0)	176 (67.2)	210 (80.2)
Questionnaires to describe your symptoms or side effects before each appointment (N=260)	18 (6.9)	12 (4.6)	29 (11.2)	37 (14.2)	164 (63.1)	201 (77.3)
Step-by-step status of your personal treatment planning while waiting at home before starting treatment (N=258)	26 (10.1)	15 (5.8)	20 (7.8)	36 (14.0)	161 (62.4)	197 (76.4)
Secure access to your personal radiotherapy treatment plan showing beam configuration and possible areas of your skin that might be affected (radiotherapy patients only; N=233)	36 (15.5)	10 (4.3)	20 (8.6)	29 (12.4)	138 (59.2)	167 (71.7)
Maps and hospital information (N=260)	22 (8.5)	23 (8.8)	32 (12.3)	35 (13.5)	148 (56.9)	183 (70.4)
Option to anonymously donate your medical data for research (N=260)	34 (13.1)	20 (7.7)	29 (11.2)	40 (15.4)	137 (52.7)	177 (68.1)
Parking information (N=255)	40 (15.7)	21 (8.2)	30 (11.8)	33 (12.9)	131 (51.4)	164 (64.3)

^a^*Pos*: the percentage of patients who rated their interest as 4 or 5, that is, the total number of patients who said they were positively “interested” in having the feature.

^b^N indicates the number of participants who answered each question. Rows are sorted by popularity of the feature offered.

Although our focus groups contained only small numbers of participants, the experience was very beneficial. They affirmed to the team that we were on the right track in terms of design, and they allowed us to make a number of changes to improve usability. A third focus group, to be held during the pilot release, will be used to inform the final release with real-world end-user feedback.

#### Element 5: Continuous Staff Input

In general, staff involvement engendered frequent discussions around 2 important points: (1) which clinical data should be accessible by patients and when and (2) the fear of increased workload by staff. These findings illustrate the importance of engaging staff early in the design and development process to ensure buy-in.

Over the course of the project, as the maturing product was presented to diverse groups, we anecdotally noticed an increase in acceptance, and ownership, of the initiative. Nevertheless, concerns about patients accessing bad news before meeting with their clinician were, and continue to be, frequently raised. Having a motivated patient colead helped ensure that the person-centeredness of the project (ie, the patient, as an equal partner in their own care, should decide for themselves what level of information they want and when) was foremost in discussions. In an attempt to account for all viewpoints, our ultimate design allows for 3 levels of information provision corresponding to those described in our patient survey (Figure 3), with each patient selecting their personal preference at the time of registration. The need to avoid increased workload on staff was identified early on by having a clinician colead the project. As such, a fundamental design component of Opal is that it should be automated. Beyond the initial setup and ongoing maintenance of rules to provide contextualized data and personalized educational material to patients, individual clinicians should notice no change to their workflows but may find that their patients are better educated on their disease and have more precise questions.

#### Element 6: End-User Testing

In total, over 60 volunteers, mainly students with mock data but including 10 patients and/or family members with access to some of their personal data, provided continuous feedback during the development process. Students mainly provided feedback regarding usability, navigation problems, and technical bugs, whereas patients mainly provided feedback regarding missing data and data presentation. Numerous improvements were, and continue to be, made as a result of user testing.

### Development Approach

Over the 3 years of software development to the pilot release, approximately 1500 commits were made to the master code base on Github by 16 unique contributors, with 39 additional branches of the patient-facing code developed by student contributors. Approximately 500 issues (bug reports) were created on Github, with 85 outstanding at the time of pilot release. These findings highlight the importance of a collaborative approach in building complex software such as a patient portal.

It was found to be difficult to find and recruit experienced full-time software developers. Being a small unknown development group within a Quebec hospital was a disadvantage when recruiting within the highly competitive marketplace for software developers in Montreal. Despite expensive advertising via the standard job-posting forums, our software developers were all ultimately recruited through word-of-mouth connections. Our access to McGill University students, in particular, paid summer students who were able to develop prototypes and prepare the groundwork for new modules within Opal, greatly facilitated our software development.

### Pilot Release

In the pilot release of Opal, patients have access to their appointment schedules, some PHI (certain radiation oncology clinical notes, laboratory test results showing longitudinal trends, and radiotherapy treatment planning status information), personalized educational material tailored to diagnosis and stage of treatment, waiting room management tools, and PRO questionnaires. Consistent with our goal of a person-centered portal, all data are contextualized with detailed explanatory content to help ensure that they are useful and empowering. Educational and explanatory content were prepared by a multidisciplinary content development team in radiation oncology that built upon existing paper-based materials. The content development team setup rules for the automated provision of education material and tags to link PHI with explanatory content. Although a Web browser version of the Opal portal is in development, it was not included in the pilot release to focus available resources.

**Figure 5:**
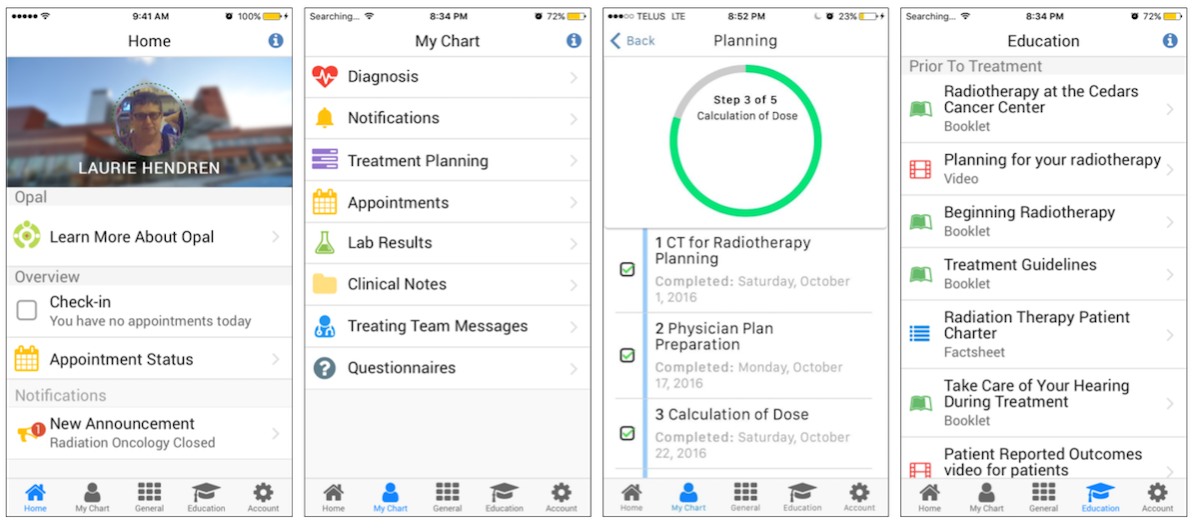
Screenshots of the Opal smartphone app provided in the pilot release, showing the Home view, the My Chart view, the radiotherapy treatment planning view, and the education material library.

**Figure 6:**
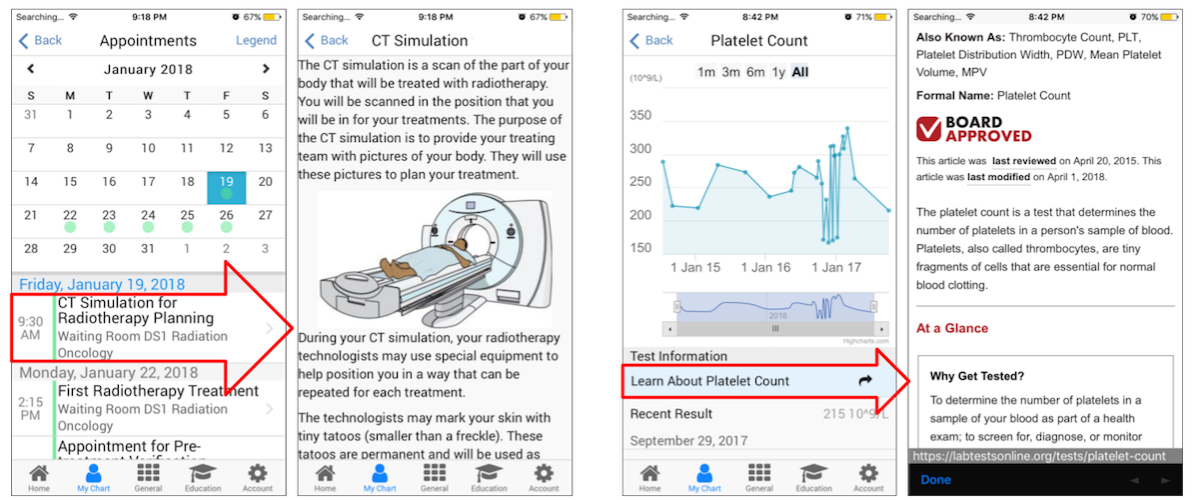
Two examples of how Opal contextualizes patient data. On the left, the Computed Tomography (CT) Simulation for Radiotherapy Planning appointment is linked to explanatory material about the CT Simulation procedure. On the right, the Platelet Count blood test results are linked to explanatory material at labtestsonline.org. The red arrows highlight the area in the left view that when tapped brings the user to the view on the right.

Screenshots of the Opal smartphone app for the pilot release are provided in Figure 5. Figure 6 illustrates how Opal provides contextualized data using 2 examples. In the first example, the patient’s calendar shows a scheduled Computed Tomography (CT) simulation appointment. A link from this appointment opens up a full explanation of what the CT simulation is. In the second example, the platelet count blood test is linked to explanatory material at Lab Tests Online [45]. Table 2 lists the features and data included in the pilot release.

Currently, Opal belongs to the members of the initial design and development team and the RI-MUHC. Ultimately, an open-source front-end code base and a licensable back-end software are envisaged.

**Table 2:** Categories of information and features or functionality identified by patients and staff to be provided to patients via Opal. The majority of information provided to patients via Opal is personalized to their disease and phase of treatment. Information made available in the pilot release is indicated.

Category (menu/tab) and features or functionality		Personalized	Pilot release
**Home screen or overview**			
	Next appointment	✓	✓
	Notifications (eg, new document and new message)	✓	✓
	Posts (messages from treating team and general hospital announcements)	✓	✓
	Status of treatment or treatment planning	✓	✓
	Waiting room management (check-in, call-in, and waiting time estimate)	✓	✓
**My chart**			
	Diagnosis information	✓	✓
	Notification archive	✓	✓
	Appointment schedule with appointment location maps	✓	✓
	Appointment change requests	✓	✗
	Treatment or treatment planning information	✓	✓
	Access to (selected) doctors’ notes and nursing notes	✓	✓
	Laboratory test results	✓	✓
	Messages from treating team	✓	✓
	Secure 2-way messaging with clinicians	✓	✗
	Patient-reported outcome and satisfaction questionnaires	✓	✓
**General information**			
	Phone directory and contact information (personalized on log-in)	✓	✓
	General hospital announcements	✗	✓
	Patient charter	✗	✓
	Parking information	✗	✓
	General hospital maps	✗	✓
	Way finding	✗	✗
	Leave feedback regarding app or portal	✗	✓
	Facility to report bugs in the app or portal	✗	✓
**Educational material (relevant and just-in-time)**			
	Videos	✓	✓
	Booklets	✓	✓
	Pamphlets or fliers	✓	✓
**Account settings**			
	Language preference	✓	✓
	Font size	✓	✓
	Synchronization with phone’s calendar	✓	✗
	Facility to update demographic information in electronic medical record	✓	✗

## Discussion

### Principal Findings

In this project, we used participatory stakeholder co-design to create a person-centered patient portal smartphone app. Our approach engaged all stakeholders by having a patient (who is also a computer scientist) colead the project as an equal with a clinician (radiation oncologist) and an informatics expert (medical physicist) and by involving patients, health care providers, technical experts, and legal personnel throughout the design and development process. Looking back over the project, we identified that we had followed 6 elements for participatory stakeholder co-design: (1) equal coleadership (patient who is also a computer scientist, clinician, and medical physicist); (2) patient preference determination; (3) security, governance, and legal input; (4) user evaluation and feedback; (5) continuous staff input; and (6) end-user testing. The benefits of participatory stakeholder co-design were clear. Our patient colead helped identify the requirements for person-centeredness, our clinical and informatics coleads strategized on their implementation within the existing hospital context, patient surveys and focus groups confirmed patient acceptability, and staff and institutional engagement ensured full stakeholder buy-in. In our estimation, the inclusion of all stakeholders in the design process is the best way to avoid the need for future redesigns and to ensure that the final product has wide acceptability.

End-user testing was achieved by the full involvement of our patient colead, partial involvement of a small cohort of volunteer tester patients who accessed some but not all of their PHI, and full involvement of a large group of enthusiastic tester students who developed and tested prototype software and accessed mock data. We found that it is important to engage end users with hands-on testing as soon as possible. We did not use wire-frame prototypes but with hindsight, we recognize that such prototyping at the beginning may have allowed us to avoid the user interface redesign provoked by our first focus group. We also maximized access to our active patient population through surveys and focus groups, and we engaged the very supportive patients’ committee of our cancer center. The wider staff of our institution were engaged continuously by means of presentations and discussions, ranging from departmental rounds to a meeting with the board of directors. We found that the legal component of the project was the most time consuming and difficult to control. If we were to start the project again, we would engage the institution’s legal team from the very beginning rather than waiting for a prototype to be ready.

True person-centeredness is difficult to achieve and, no doubt, continuous refinements to our software to ensure its person-centeredness will be required in future. Fundamental to achieving this is the involvement of patients now and into the future. Our attempt to maximize person-centeredness in our smartphone app includes 3 important considerations: (1) patients should decide for themselves their level of PHI access, (2) all PHI provided to patients should be contextualized with explanatory content so that they are useful and empowering, and (3) educational material should be personalized and tailored to the patient’s immediate medical situation. These considerations are consistent with the findings of a study on patients’ experience with cancer published recently by the Canadian Partnership Against Cancer [46].

The concerns of staff regarding increased workload because of the presence of a patient portal were addressed by designing the software to be automated. By using a rule-based system to send educational material and explanatory content to patients, a once-off setup is required by staff in each clinical setting. Thus, beyond the need for regular maintenance, the ongoing portal-related workload for staff should be minimal. Our multidisciplinary content development team in radiation oncology took care of the initial setup for Opal’s pilot release. The team met for several hours each week for about 3 months to prepare the content. Patient sign-up for Opal during its pilot release is facilitated by staff from our cancer center’s foundation. Ultimately, for a hospital-wide portal, sign-up should be facilitated by the hospital’s admitting staff and may require additional personnel.

### Comparison With Prior Work

It is well established that user involvement is a necessary component of successful software design [33]. Similarly, it is increasingly recognized that co-design is a necessary ingredient for successful quality improvement in health care [34-36]. Taken together, it is clear that for development of patient-facing software to be successful, all stakeholders, including patients and health care providers, must be involved in the design process. Achieving meaningful stakeholder involvement in practice is often difficult; however, clinicians are busy and may not have time to provide sufficient feedback, and active patients, although plentiful, are often not easy for development teams to access for reasons of confidentiality.

Our work, qualitatively and quantitatively, has shown that patients overwhelmingly feel that digital access to their PHI is important, with a majority of patients desiring immediate access to all of their data. These findings are consistent with experience reported elsewhere [47-49]. Our clinical staff, although supportive of the Opal initiative in general, tended to raise concerns that patients accessing their PHI in the absence of a clinician to provide support may result in anxiety and misunderstandings. Again, these findings are consistent with the literature [50-52]. Evidence from early adopter patient portals, however, tends to show that clinicians need not be too concerned [53]. For example, a pilot study on providing laboratory test results to patients in British Columbia found no difference in the levels of anxiety among patients who received their laboratory results online compared with a control group [54]. Similarly, early results from 7 clinics using the *myUHN* patient portal at the University Health Network in Toronto indicate no significant evidence of increased patient anxiety [55]. Of particular interest to our project in the context of cancer care, where laboratory results may contain alarming information, qualitative studies by Rexhepi et al [56] and Giardina et al [51] found that the majority of patients in their studies wanted immediate online access to their test results even if those results were alarming. In Rexhepi et al’s [56] study, many patients expressed the belief that immediate access causes less anxiety than having to wait to discuss with a physician regardless of the nature of the results. These studies and others, for example, Ammenwerth et al’s [11], have also shown that online access to PHI enables patients to better prepare for their consultations, which in turn improves clinician-patient communication, a goal of person-centered care. Throughout our participatory stakeholder co-design process, we found it useful to highlight these findings from other patient portal projects. Indeed, as our project matured, we noticed a softening in attitudes regarding sharing PHI with patients via Opal, a change we attribute to the involvement of all stakeholders in our design and development process.

### Limitations

An important limitation of our quantitative findings is that they are specific to our context—a comprehensive cancer center in Montreal. For example, our convenience-sampled patient survey was solely intended to provide input to the design and development team. As such, our quantitative results may contain biases to our setting that limit wider applicability. Our qualitative findings (ie, our description of the approach followed) are based on our retrospective analysis of the design and development strategy that worked for us. Another team following a broadly similar approach may describe it differently.

### Conclusions and Future Work

We have designed and developed a patient portal smartphone app from within the Quebec health care system using a participatory stakeholder co-design approach, involving patients, staff, software developers, and students. Our methodology consisted of 6 core elements: (1) equal coleadership (patient who is also a computer scientist, clinician, and medical physicist); (2) patient preference determination; (3) security, governance, and legal input; (4) continuous user evaluation and feedback; (5) continuous staff input; and (6) end-user testing. Our final design adhered to principles of person-centeredness, recognizing that patients should decide for themselves their level of PHI access, all PHI should be contextualized with explanatory content, and educational material should be personalized and timely. As our project matured, and more and more stakeholders were engaged, we noticed an increase in the acceptance by clinical staff of the concept of sharing PHI with patients.

Future work will focus on evaluating the uptake and acceptability of our portal during its pilot release and expansion of its use beyond our cancer center to our general hospital and to additional health care institutions.

## Appendix

Multimedia Appendix 1 Focus group guidelines document. Used by the moderators of the first patient focus group that evaluated the early Opal prototype app.

Multimedia Appendix 2 Elements of person-centeredness, clinician acceptability, and informatics feasibility identified during the development of Opal and facilitated by the stakeholder co-design approach that was followed.

Multimedia Appendix 3 Security and governance recommendations for development of a patient portal as identified by our institution's Security and Governance team.

## Abbreviations

CT: Computed Tomography
eHealth: electronic health
EMR: electronic medical record
MUHC: McGill University Health Centre
PHI: personal health information
PRO: patient-reported outcome
RI-MUHC: Research Institute-MUHC
