# Supplementary material for: Design and Development of a Person-Centered Patient Portal Using Participatory Stakeholder Co-Design
Source: J Med Internet Res. 2019 Feb 11;21(2):e11371. doi: 10.2196/11371 (PMC6388099; doi:10.2196/11371)
Supplement: Multimedia Appendix 1 [file jmir_v21i2e11371_app1.pdf]

Opal Patient Focus Group

Focus Group Guide for Team Members

Note: this document, intended for the staff who helped moderate the focus group, has been redacted to remove names and certain details to allow for dissemination to a larger audience.

**Date and time**	Details removed
**Moderator(s)**	Names removed
**Team members**	Names removed
**Room**	Details removed
**Patient Volunteers**	Names removed
**Catering requirements**	Tea and coffee, some fruit and cookies will be provided.
**AV requirements**	Large digital display screen showing Opal<br>Whiteboard and markers
**Handouts**	List of questions available to everyone
**Brief summary of focus group and questions**	1. We will start off with some welcome messages and introductions of the team members.<br>2. The patients will be asked to introduce themselves (when they had their radiotherapy, what they do for a living and how they found out about this focus group).<br>3. The moderators will briefly explain the Opal development team and the goals of the Opal project. **At this point, so as not to bias the participants, we should not say what we will or can/cannot provide via the patient portal.**<br>4. **Question 1:** What sort of information do you think might be useful for patients to receive via an app/portal? It can be anything at all that patients might want. Ask participants to imagine that there are no restrictions - legal, technical or otherwise.<br>5. **Question 2:** What information did you find hardest to get while you were a radiotherapy patient?<br>6. **In-hand app demo:** At this point we will need phones showing the prototype app and we will ask the patient volunteers to try it out. We won't help them with it. They need to open it from scratch and navigate through it.

	7. Each team member should work with one patient on this part and the team member should try to answer the following questions:<br>○ Was the patient able to navigate the app easily?<br>○ What was most confusing feature for the patient?<br>○ What impressed the patient most?<br>○ What did the patient spend the most time looking at?<br>○ Did the patient say “wow!” on any feature(s)? Which?<br>○ Did the patient say “this would have been so useful” for any feature(s)? Which?<br>○ Did the patient say “it would be nice if this also did that” on any feature? Which?<br>8. **Large screen demo:** Here we will do a final demo of the app on the large screen and go through each of the features and ask the patients to comment on each feature:<br>○ I don’t want to see this<br>○ Not needed<br>○ Not needed but nice<br>○ Useful<br>○ Very useful<br>○ Extremely useful - I wish I had had this when I was having my radiotherapy

Team Member Worksheet

Question	Answer(s)
**Question 1:** What sort of information do you think might be useful for patients to receive via an app, in addition to waiting time information?<br><br>It can be anything at all that patients might want. Imagine that there are no restrictions - legal, technical or otherwise.	
**Question 2:** What information did you find hardest to get while you were a radiotherapy patient?	
Was the patient able to navigate the app easily?	
What was most confusing feature for the patient?	
What impressed the patient most?	
What did the patient spend the most time looking at?	
Did the patient say “wow!” on any feature(s)? Which?	
Did the patient say “this would have been so useful” for any feature(s)? Which?	
Did the patient say “it would be nice if this also did that” on any feature? Which?	

Opal Patient Focus Group

TV Screen Demo Worksheet

1. I don't want to see this
2. Not needed
3. Not needed but nice
4. Useful
5. Very useful
6. Extremely useful - I wish I had had this when I was having my radiotherapy

**Feature**	**Score**
Welcome message	1 2 3 4 5 6
Status	1 2 3 4 5 6
Last Appointment/check-in	1 2 3 4 5 6
Navigation tool, top left	1 2 3 4 5 6
Check-in feature	1 2 3 4 5 6
Contacts	1 2 3 4 5 6
Notifications	1 2 3 4 5 6
Appointments	1 2 3 4 5 6
Appointments list	1 2 3 4 5 6
Appointments calendar	1 2 3 4 5 6
Treatment plan	1 2 3 4 5 6
Documents	1 2 3 4 5 6
Consultation Note	1 2 3 4 5 6
Radiotherapy treatment plan	1 2 3 4 5 6
End of treatment note	1 2 3 4 5 6
Messages	1 2 3 4 5 6
Educational material	1 2 3 4 5 6
Booklet	1 2 3 4 5 6
Video	1 2 3 4 5 6

Diagnosis-specific	1	2	3	4	5	6
When radiotherapy ends	1	2	3	4	5	6
Feature to get these educational materials at the point in time when most appropriate	1	2	3	4	5	6
Maps	1	2	3	4	5	6
Appointment location on map	1	2	3	4	5	6
Notes	1	2	3	4	5	6
Account settings	1	2	3	4	5	6
Allow an alias for the screen display	1	2	3	4	5	6
	1	2	3	4	5	6
	1	2	3	4	5	6

Takeaway Letter for Patient Participants

Dear focus group participant,

Thank you for your time and insight as we develop our mobile phone app for Radiation Oncology patients at the Cedars Cancer Centre.

We appreciate all comments and feedback. If you think of anything you would like to share with us after the focus group, please email us at **xxxxx**.

The demo version of the app is online at **opalmedapps.com** *(Note: We have updated this URL, since the URL available at the time of the focus group is no longer active and has been replaced with this one.)*. Please feel free to share it with your family and friends. We welcome their comments and feedback also.

If you would like to be involved in future similar focus groups at the Cedars Cancer Centre or the MUHC please let us know.

Thanks again,

The Opal Development Team
