# Supplementary material for: Design and Development of a Person-Centered Patient Portal Using Participatory Stakeholder Co-Design
Source: J Med Internet Res. 2019 Feb 11;21(2):e11371. doi: 10.2196/11371 (PMC6388099; doi:10.2196/11371)
Supplement: Multimedia Appendix 2 [file jmir_v21i2e11371_app2.pdf]

Multimedia Appendix

Multimedia Appendix 1. Elements of person-centeredness, clinician acceptability, and informatics feasibility identified during the development of Opal and facilitated by the stakeholder co-design approach that was followed.

Element	Examples
Person-centeredness	Inclusion of patient-identified features and functionality (e.g. view of progress in radiotherapy treatment planning process, direct phone numbers with extension-dialing built-in)
	Level of PHI provision chosen by patient
	Active features (e.g. push notifications, waiting room management tools, patient-reported outcome and patient-satisfaction questionnaires)
	Contextualized PHI (e.g. links to explanations of blood tests, clinical notes)
	Useful/actionable data (e.g. appointment-specific preparation advice, ability to print or electronically share data)
	Multi-specialty data (e.g. all appointments, all clinical notes, regardless of source specialty within institution)
	Connected data (e.g. longitudinal view of blood test trends as opposed to just individual results)
	Navigation aids (e.g. maps, hospital information, etc.)
	Personalized and just-in-time educational material
Clinician-acceptability	Automation of PHI provision - no increased workload for staff
	Automation of educational material provision - no increased workload for staff
	Provision of clinical notes only after clinician sign-off
Informatics feasibility	Use of existing databases only
	Generalized modular approach - expandable to any specialty
	Vendor-agnostic approach using institution's interface engine
	Privacy by design
