# Supplementary material for: Design and Development of a Person-Centered Patient Portal Using Participatory Stakeholder Co-Design
Source: J Med Internet Res. 2019 Feb 11;21(2):e11371. doi: 10.2196/11371 (PMC6388099; doi:10.2196/11371)
Supplement: Multimedia Appendix 3 [file jmir_v21i2e11371_app3.pdf]

Multimedia Appendix 2. Security and governance recommendations for development of a patient portal as identified by our institution's Security and Governance team.

**Area of Concern**	**Recommendation**
Privacy and confidentiality of PHI	Security risk fully explained and disclosed to patient
	Reminder to patient of need to secure his/her device at each login
	Automatic logout after five minutes of inactivity
	Secure sandboxing of data on user's device and deletion on logout
	Use of security questions for second level of authentication
	Final approval required from institution's senior management
	Release of PHI coordinated with institution's Medical Records team
	Requirement for technical vulnerability assessment pre-release
Maintenance of service	Development of a service interruption plan-of-action
	Development of a policy and procedure manual for the complete solution
	Development of an end-user manual for all staff-facing software
	Full documentation for the software (patient and staff-facing)
	Separation of development, test, and production environments
	Failover and redundancy solution and regular tests
Compliance	User logging and activity monitoring
	Re-assessment of all security and governance aspects in case of major software changes
